# Molecular Detection of Toxigenic *Clostridioides difficile* among Diarrheic Dogs and Cats: A Mounting Public Health Concern

**DOI:** 10.3390/vetsci8060088

**Published:** 2021-05-22

**Authors:** Ahmed Samir, Khaled A. Abdel-Moein, Hala M. Zaher

**Affiliations:** 1 Department of Microbiology, Faculty of Veterinary Medicine, Cairo University, Cairo 12211, Egypt; ahmed.samir@cu.edu.eg; 2 Department of Zoonoses, Faculty of Veterinary Medicine, Cairo University, Cairo 12211, Egypt; khal_105@cu.edu.eg

**Keywords:** toxigenic *C. difficile*, dogs, cats, public health

## Abstract

Nowadays, pet animals are known to be asymptomatic carriers of *Clostridioides* *difficile*. This study was conducted to investigate the burden of toxigenic *C. difficile* among diarrheic dogs and cats using direct PCR on fecal samples to reveal better insights about the epidemiology of such toxigenic strains referring to its public health significance. For this purpose, fecal samples were obtained from 58 dogs and 42 cats experiencing diarrhea. Following DNA extraction, the extracted DNA was examined for the occurrence of *C. difficile* as well as toxigenic strains through the detection of *C. difficile* 16S rRNA and toxin encoding genes (*tcdA*, *tcdB*, *cdtA* and *cdtB*) using PCR. Moreover, partial DNA sequencing of toxigenic strains retrieved from dog and cat was carried out. Of 100 examined diarrheic animals, 90 (90%) were *C. difficile* positive, including 93.1% and 85.7% of dogs and cats, respectively. In addition, toxigenic strains were detected in 13 animals, giving an overall prevalence 13% with the following prevalence rates among dogs and cats 12.1% and 14.3%, respectively. Furthermore, the phylogenetic analysis of the obtained sequence revealed high genetic relatedness of *tcdA* sequence obtained from a cat to strains of human diarrheic cases to point out the public health threat of such sequence. In conclusion, the direct detection of toxigenic *C. difficile* using PCR among dogs and cats highlights the potential role of household pets as a source for such strains to human contacts.

## 1. Introduction

*Clostridioides difficile* (formerly known as *Clostridium difficile*) is an emerging enteric pathogen in human and veterinary medicine [1]. It is a Gram-positive, strictly anaerobic, spore forming toxin-mediated bacillus [2]. In the last four decades, after admission of broad-spectrum antibiotics, the role of *C. difficile* in enteric diseases began to flare up to become a remarkable cause of nosocomial associated diarrhea and pseudomembranous colitis among human being [3]. However, nowadays, there is increasing number of *C. difficile* infection (CDI) cases outside health care settings referring to community acquired CDI, which accounts for one quarter of all reported CDI cases [4,5]. Nonetheless, there is no definitive source of CDI in the community settings [6] and this has urged researchers to investigate the potential role of animals as a vector for transmission of CDI [4]. Notably, this pathogen has been implicated in gastrointestinal diseases among diverse animal species, including food producing animals as well as companion animals [7,8,9,10,11]. Regarding pet animals, there were a lot of reports investigated *C. difficile* in dogs with gastrointestinal disorders [12,13,14,15] while in cats, little is known concerning association between *C. difficile* and feline enteric diseases [16,17]. The characteristic diarrhea and gastrointestinal tract inflammation in pet animals and humans are mainly attributed to toxin producing *C. difficile* [18,19]. Basically, pathogenic *C. difficile* strains produce two main toxins: toxin A and toxin B which encoded by *tcdA* and *tcdB* genes, respectively [20] with some strains producing a binary toxin *C. difficile* transferase (CDT) [21]. While toxin A is an enterotoxin causing severe gut inflammation, toxin B is a potent cytotoxin that is responsible for cellular death and damage of epithelial tissue [22]. Investigation of *C. difficile* and its toxins in diarrheic animals relies on conventional methods such as culture may yield underestimated results [13,23]. Recently, direct detection of *C. difficile* in animal fecal samples using PCR was found to give significantly higher detection rate rather than conventional culture technique [24] whereas, the direct detection of toxin encoding genes is a reliable tool for the detection of toxigenic strains [25]. Accordingly, the current study was carried out to investigate the occurrence of toxigenic *C. difficile* via direct PCR on feces of pet animals suffering from diarrhea to give insight about the burden of toxigenic *C. difficile* strains among diarrheic pet animals for better understanding the epidemiology of such strains referring to its public health implication.

## 2. Materials and Methods

### 2.1. Ethical Statement

The protocol of this study was approved by ethical committee of Faculty of Veterinary Medicine, Cairo University, Egypt with an ethical approval code: Vet CU28/04/2021/321.

### 2.2. Sample Collection

Fecal samples were obtained from 100 diarrheic pet animals (58 dogs and 42 cats) from private veterinary clinics where animals of different ages were included in this study. These samples were collected in sterile cups, transported in an icebox to the laboratory and stored at −20 °C for further processing.

### 2.3. Molecular Investigation of C. difficile and Toxin Encoding Genes

#### 2.3.1. DNA Extraction

DNA was extracted from each fecal sample using FavorPrep™ Stool DNA Isolation Mini Kit (Favorgen, Taiwan, Cat No. FASTI 001-1) according to the manufacturer protocol. Then after, the extracted DNA was stored at −20 °C till further molecular analysis.

#### 2.3.2. Direct Detection of *C. difficile*

**Figure 1:**
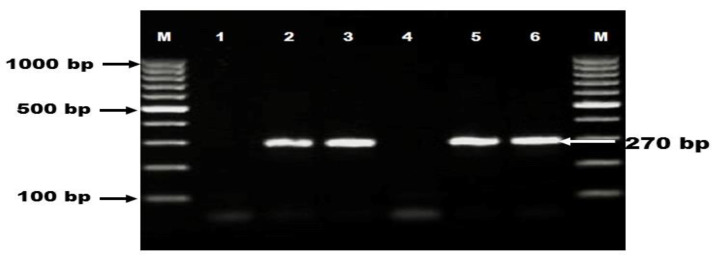
Occurrence of *C. difficile* 16S rRNA among diarrheic dogs and cats. Lane M: DNA ladder (100 bp); lane 1: negative control; lanes 2, 3, 5, 6: positive samples with specific band at 270 bp; lane 4: negative sample.

The extracted DNA was screened for the presence of *C. difficile* via direct detection of *C. difficile* 16S rRNA using the following primers: B (CCGTCAATTCMTTTRAGTTT) and PG-48 (CTCTTGAAACTGGGAGACTTGA) (Metabion, Steinkirchen, Germany) [26]. The PCR reaction was carried out in a final volume 25 μL where 3 μL of DNA template, 1 μL of each primer, 12.5 μL of Cosmo PCR red master mix (Willowfort, Birmingham, UK, Cat No. WF10203001) and 7.5 μL of nuclease free water were included in each reaction. The thermal profile of PCR reaction was as follows: Initial denaturation at 95 °C for 3 min followed by 40 cycles of denaturation at 95 °C for 30 s, annealing at 44 °C for 30 s, extension at 72 °C for 30 s then final extension at 72 °C for 5 min. Afterwards, amplicons were analyzed with agarose gel electrophoresis (BioRad, Hercules, USA) and photographed to yield specific band at 270 bp (Figure 1).

#### 2.3.3. Direct Detection of *C. difficile* Toxin Genes

##### *tcdA* and *tcdB* Genes

**Figure 2:**
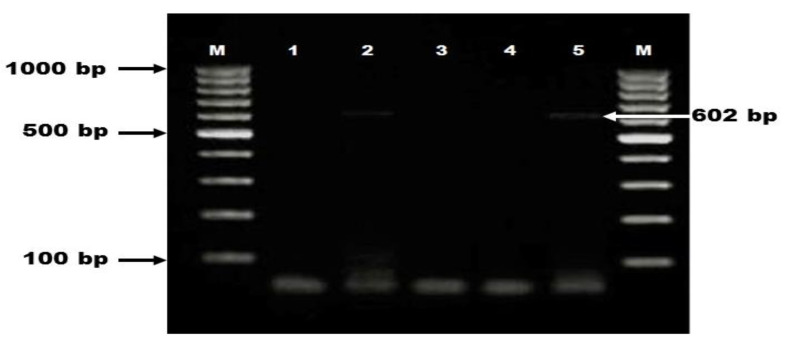
Molecular detection of *C. difficile tcdA* gene among diarrheic dogs and cats. Lane M: DNA ladder (100 bp); lane 1: negative control; lanes 2,5: positive samples showed specific band at 602 bp; lanes 3,4: negative samples.

Investigation of *C. difficile tcdA* and *tcdB* genes encoding toxin A and toxin B, respectively was carried out in all animals. Primers designed to amplify regions of *tcdA* and *tcdB* were as follow: *tcdA* (YT-28 GCATGATAAGGCAACTTCAGTGG and YT-29 GAGTAAGTTCCTCCTGCTCCATCAA), *tcdB* (YT-17 GGTGGAGCTGCTTCATTGGAGAG and YT-18 GTGTAACCTACTTTCATAACACCA) (Metabion, Steinkirchen, Germany) [27]. The PCR assay was done at 95 °C for 3 min then 40 cycles of denaturation (95 °C for 20 s), annealing (53 °C, 49 °C for *tcdA* and *tcdB* respectively for 25 s), extension (72 °C for 1 min) followed by final extension at 72 °C for 7 min. The PCR products were observed under UV transilluminator (BioRad, Hercules, CA, USA) after electrophoresis step in 1.5% agarose gel (Sigma-Aldrich, Saint Louis, USA, Cat No. A0576) stained with 0.5 μg/mL of ethidium bromide (Sigma-Aldrich, Saint Louis, USA, Cat No. E7637) as specific band of *tcdA* gene was showed at 602 bp (Figure 2).

##### *cdtA* and *cdtB* Genes

The multiplex PCR amplification for binary toxin genes (*cdtA* and *cdtB*) was carried out as follows: after 4 min of initial denaturation at 94 °C, 30 cycles of 94 °C for 45 s, 52 °C for 1 min and 72 °C for 80 s were conducted then followed by 72 °C for 5 min [28].

#### 2.3.4. Partial DNA Sequencing of *C. difficile tcdA* and *tcdB* Genes

One PCR product of toxin A obtained from a cat and another one of toxin B retrieved from a dog were purified via a QIAquick purification kit (Qiagen, Hilden, Germany, Cat No. 28104) then they were subjected for sequencing using Big Dye Terminator V3.1 kit (Thermo Fisher, Waltham, MA, USA, Cat No. 4337455) in ABI 3500 Genetic Analyzer (Applied Biosystems, Foster City, CA, USA).

### 2.4. Nucleotide Sequence Accession Numbers

Partial sequences of *C. difficile tcdA* and *tcdB* genes were submitted to GenBank and deposited in GenBank database with the following accession numbers: MW340088 for *tcdA* and MW357902 for *tcdB*.

### 2.5. Sequence Identity BLAST Analysis

The obtained *tcdA* and *tcdB* sequences from cat and dog respectively, were compared with *C. difficile* strains available on GenBank using NCBI website via BLAST analysis to display the identity percentage between our sequences and those of human clinical cases from different countries to clarify the public health significance of such strains.

### 2.6. Phylogenetic Analysis

**Figure 3:**
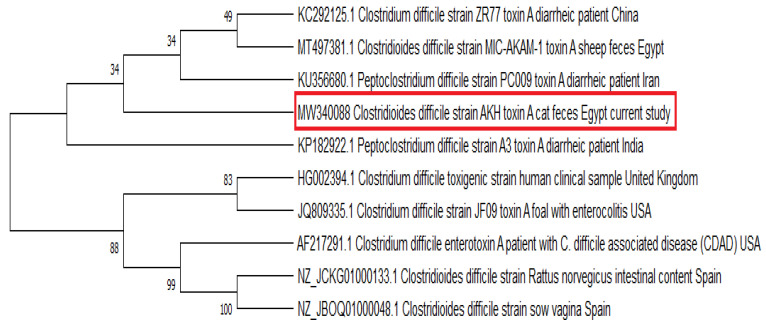
Phylogenetic consensus tree was constructed using neighbor-joining approach via Mega 7 software to display the genetic relatedness between *tcdA* sequence obtained from a cat and those retrieved from Genbank.

The recovered *tcdA* cat strain was aligned against similar *C. difficile* toxin A sequences retrieved from animals as well as strains obtained from human clinical cases worldwide to confer the genetic relatedness between pets and human strains to understand the public health implications of our findings. Clustal W multiple alignment was conducted using Bioedit software version 7.0.9 while MEGA 7 software was used to construct phylogenetic tree via neighbor-joining approach where bootstrap consensus tree was obtained with 500 replicates (Figure 3).

### 2.7. Statistical Analysis

The influence of age on prevalence rate of toxigenic *C. difficile* was analyzed by SPSS software version 18.0 using chi square (χ2) test. The result was considered statistically significant when *p*-value was less than 0.05.

## 3. Results

Of 100 examined diarrheic animals, 90 (90%) were *C. difficile* positive, including 93.1% and 85.7% of dogs and cats, respectively. For toxigenic *C. difficile*, 13 (13%) out of 100 animals had *C. difficile* toxins comprising 12.1% (7/58) and 14.3% (6/42) of dogs and cats, respectively. Moreover, according to toxin production type, 4 dogs and 4 cats were positive for *tcdA* and negative for *tcdB* while one dog and two cats carried toxin B and negative for toxin A as well as both *tcdA* and *tcdB* genes had been detected in two dogs. On the other hand, none of binary toxins (*cdtA* and *cdtB*) was found among the examined dogs and cats as shown in Table 1. Regarding animal age, the prevalence of toxigenic *C. difficile* was as follows: 14% (less than 6 months), 14.8% (6–12 months) and 16.7% (greater than 12 months) (Table 2). Statistically, no significant relationship (*p* value = 0.94) was observed between toxigenic *C. difficile* and animal age. The similarity ratios between the obtained *C. difficile tcdA* and *tcdB* sequences in this study and those of public health importance according to the BLAST analysis were displayed in Table 3.

## 4. Discussion

In the last few years, pet dogs and cats were found to be potential reservoirs for some emerging nosocomial pathogens with great public health concern [29,30]. Such previous studies have paved the way for more investigations about the role of pet animals in the epidemiology of other nosocomial pathogens likewise *C. difficile* and nowadays, the implication of household pets in community acquired CDI is an ongoing public health issue [6]. In the current study, *C. difficile* was detected in 90% of diarrheic pet animals where 93.1% and 85.7% of dogs and cats were positive, respectively. Our results were higher than those reported in previous studies 6.7% [15], 25% [31] for dogs and 12.9% [17], 15.7% [31] for cats suffering from diarrhea. Such high unexpected results in the current study may be owed to the direct detection of *C. difficile* by PCR using 16S rRNA primers can detect as little as 10 cells of *C. difficile* among 10^10^–10^11^ total bacterial cells per one gram of stool [32]. On the contrary, other studies recovered *C. difficile* via conventional culture technique which needs at least 1000 cfu/gram of feces on the selective *C. difficile* culture media to yield successful cultivation. Therefore, the direct detection of *C. difficile* by PCR can elucidate the burden of CDI among diarrheic pet animals and consequently triggers a growing public health concern.

Regarding toxigenic *C. difficile*, 13 out of 100 diarrheic pet animals were positive for toxin encoding genes, whereas 12.1% (7/58) of investigated dogs carried *C. difficile* toxins. Our finding was lower than that reported by Weese et al. [12] who found 21% (18/87) of examined diarrheic dogs were toxigenic using an ELISA assay. While in cats, the prevalence of toxigenic *C. difficile* was 14.3% (6/42). Such result was higher than that reported by Silva et al. [17] who detected toxigenic *C. difficile* in 3 (4.3%) out of 70 diarrheic cats by PCR carried out on recovered *C. difficile* isolates.

From a public health point of view, *C. difficile* associated diarrhea in human being is mainly attributed to toxigenic strains [19]. Importantly, in the current study, there were two dogs carried both toxin A and toxin B which may refer to presence of A+/B+ toxinotype. *C. difficile* A+/B+ is the most predominant toxigenic strain isolated from diarrheic dogs in studies conducted by Wetterwik et al. [13], Andrés-Lasheras et al. [15], Ghavidel et al. [33] and Silva et al. [34]. Likewise, it is the most pathogenic *C. difficile* toxinotype in human being and is primarily accounted for *C. difficile* associated disease (CDAD) worldwide [35,36]. Moreover, there were one dog and two cats had *tcdB* gene but negative for *tcdA* which may indicate A−/B+ strain. Such toxinotype has attracted the attention of researchers in recent years [37] because it has been incriminated in four nosocomial outbreaks of *C. difficile* associated diarrhea in Canada [38], Netherlands [39], Japan [40] and Dublin [41] as well as 4 dogs and 4 cats were found to be toxin A positive and toxin B negative; this strain also had been detected among diarrheic patients in intensive care unit [42]. On the other hand, all the examined dogs and cats were negative for binary toxin genes. In agreement with our result, Andrés-Lasheras et al. [15] and Silva et al. [17] who could not find binary toxins in *C. difficile* isolates recovered from diarrheic dogs and cats, respectively. Pet dogs and cats are in frequent and close contact with their owners and usually share the same places at home like living room and bedroom. Accordingly, diarrheic pet animals may be considered as a potential source for dissemination of toxigenic *C. difficile* strains within a community. Therefore, our findings indicate that the direct PCR detection of toxigenic *C. difficile* in feces of diarrheic dogs and cats can give a better insight to understand the epidemiology of toxigenic *C. difficile* infection among pet animals.

On the other hand, the prevalence of toxin producing *C. difficile* was found to be increased with age of pet animals as animals of age greater than 12 months had a higher percentage (16.7%) but there was no significant relationship between age and prevalence rate of toxigenic *C. difficile*. Similarly, Álvarez-Pérez et al. [23] and Diniz et al. [43] reported that shedding of *C. difficile* and its toxins was increased with pet animals of higher ages.

Interestingly, in this study, we provide *C. difficile tcdA* and *tcdB* partial sequences from a cat and a dog, respectively, where cat and dog strains showed high identity percentage of 99.81%–100% and 99.43%–99.72% respectively to *C. difficile* isolates retrieved from patients with diarrhea and pseudomembranous colitis worldwide to highlight the public health impact of such strains. In the meantime, phylogenetic tree was constructed to encompass *C. difficile* toxin A sequences from animals as well as human strains including diarrheic patients from different countries (Figure 3). It was obvious that *tcdA* sequence from a cat was grouped within the same cluster with that reported in sheep in the same country (Egypt) and those of human diarrheic cases originated from Asian countries (China, India, and Iran). Thus, the high genetic relatedness of our sequence to those of humans points out a potential relationship between cats and diarrheal infection in human being rendering pet animals a potential zoonotic source for toxigenic *C. difficile* human infection.

## 5. Conclusions

This study provides more knowledge regarding the epidemiology of toxigenic *C. difficile* infection among diarrheic dogs and cats. Remarkably, the direct detection of toxigenic *C. difficile* using PCR in animal samples opens a gate for better assessment of toxigenic CDI burden among household pets which subsequently, reflects on human health.

## Figures and Tables

**Table 1:** Occurrence of *C. difficile* 16S rRNA and toxin encoding genes among diarrheic dogs and cats.

Animal Species	No. of Examined Animals		No. of Positive Animals (%)				
		*C. difficile* 16S rRNA		Toxigenic *C. difficile*			
			*tcdA+tcdB-*	*tcdA-tcdB+*	*tcdA+tcdB*+	Binary Toxins (*CDT*)	Total
Dogs	58	54 (93.1)	4(6.9)	1 (1.7)	2 (3.4)	0 (0)	7 (12.1)
Cats	42	36 (85.7)	4 (9.5)	2 (4.8)	0 (0)	0 (0)	6 (14.3)
Total	100	90 (90)	8 (8)	3 (3)	2 (2)	0 (0)	13 (13)

**Table 2:** Occurrence of toxigenic *C. difficile* among pet animals of different ages.

Age of Animals	No. of Examined Animals	Positive Animals	
		No.	%
<6 M	43	6	14
6–12 M	27	4	14.8
>12 M	30	5	16.7
Total	100	15	15

**Table 3:** The identity percentage of obtained *C. difficile tcdA* and *tcdB* partial sequences in this study with *C. difficile* strains deposited in Genbank of public health significance.

Sequence	Genbank ID	Isolation Source	Country	% Identity
MW340088 (*tcdA* cat sequence)	KP182922.1	Diarrheic patient	India	100
	CP022524.1	Hospitalized pediatric patient with diarrhea	USA	99.81
	CP010905.2	Patient with severe pseudomembranous colitis	Switzerland	99.81
	KC292061.1	Diarrheic patient	China	99.81
MW357902 (*tcdB* dog sequence)	DQ117266.1	Patient with antibiotic associated diarrhea	France	99.72
	KC292138.1	Diarrheic patient	China	99.48
	CP010905.2	Patient with severe pseudomembranous colitis	Switzerland	99.48
	DQ117268.1	Patient with pseudomembranous colitis	France	99.43

## Data Availability

Not applicable.
